# Phytonutrient diet supplementation promotes beneficial Clostridia species and intestinal mucus secretion resulting in protection against enteric infection

**DOI:** 10.1038/srep09253

**Published:** 2015-03-19

**Authors:** Marta Wlodarska, Benjamin P. Willing, David M. Bravo, B. Brett Finlay

**Affiliations:** 1 Michael Smith Laboratories, University of British Columbia, Vancouver, BC, V6T 1Z4; 2 Department of Microbiology and Immunology, University of British Columbia, Vancouver, BC, V6T 1Z4; 3 Department of Biochemistry and Molecular Biology, University of British Columbia, Vancouver, BC, V6T 1Z4; 4 Department of Agricultural, Food and Nutritional Science, University of Alberta, Edmonton AB, T6G 2P5; 5 Pancosma, Geneva, Switzerland

## Abstract

Plant extracts, or phytonutrients, are used in traditional medicine practices as supplements to enhance the immune system and gain resistance to various infectious diseases and are used in animal production as health promoting feed additives. To date, there are no studies that have assessed their mechanism of action and ability to alter mucosal immune responses in the intestine. We characterized the immunomodulatory function of six phytonutrients: anethol, carvacrol, cinnamaldehyde, eugenol, capsicum oleoresin and garlic extract. Mice were treated with each phytonutrient to assess changes to colonic gene expression and mucus production. All six phytonutrients showed variable changes in expression of innate immune genes in the colon. However only eugenol stimulated production of the inner mucus layer, a key mucosal barrier to microbes. The mechanism by which eugenol causes mucus layer thickening likely involves microbial stimulation as analysis of the intestinal microbiota composition showed eugenol treatment led to an increase in abundance of specific families within the Clostridiales order. Further, eugenol treatment confers colonization resistance to the enteric pathogen *Citrobacter rodentium*. These results suggest that eugenol acts to strengthen the mucosal barrier by increasing the thickness of the inner mucus layer, which protects against invading pathogens and disease.

The animal production industry commonly supplements livestock feed with subtherapeutic doses of antibiotics to prophylactically prevent bacterial infections and, importantly, to promote growth of the animals^1^,^2^. This constant antimicrobial exposure results in the selection of antibiotic-resistant microbes (both pathogenic and commensal) within the animals^3^. Humans are then exposed to these antibiotic-resistant microbes through direct animal contact, contact with soil and water contaminated with animal waste, and the consumption or handling of food products^2^. This transfer of antibiotic-resistant microbes to humans has great consequences to health as many of the antibiotics or classes of antibiotics are also used to treat clinical infections in humans, making certain infectious diseases difficult to cure^4^. This controversial practice of antibiotic use in livestock feed is still in use in North America but is banned in many countries around the world, including those belonging to the European Union^2^. In order to compete with animal production practices in North America alternate strategies must be developed to promote animal health and growth comparable to what is gained with antibiotic use.

A variety of plant extracts, or phytonutrients, are used in traditional medicine practices as supplements to enhance the immune system and gain resistance to various infectious diseases. Prophylactic treatment of rats with carvacrol and eugenol was shown to be sufficient to protect against *Candida albicans* infection^5^. Both carvacrol and eugenol have been shown to possess anti-bacterial activity against *Escherichia coli*^6^. Furthermore, eugenol was found to alter the integrity of the bacterial membrane^7^ as well as inhibit quorum sensing^8^. Current research has also shown efficacy of the use of phytonutrients as feed supplements for animal production, as they have been shown to enhance immunity gene expression and promote growth^9^. Low dietary doses of phytonutrients alone or in combination conferred protection to different farm animals. This was observed in broilers after infection with different *Eimeria* species^10^,^11^,^12^ as well as in piglets infected with an enteropathogenic *E coli*^13^ or the virus of porcine pulmonary and reproductive syndrome^14^.

The protective effect of phytonutrients against infection is becoming evident as well as their effect on animal growth but information is still lacking on the mechanisms driving these effects. Evidence thus far indicates that phytonutrients likely have direct effects on host physiology and the immune system, along with indirect effects mediated by changes in the microbial communities within the intestine. The ability of a phytonutrient to alter intestinal homeostasis and microbial composition depends on its chemical characteristics including bioavailability, utilization as metabolic substrate by microbes, and antimicrobial activity^15^. We hypothesized that phytonutrients can stimulate both immune and microbial factors to protect against infection, possibly by altering the microbial architecture of the intestine to promote expression of innate immune factors, including stimulating mucus secretion. In the large intestine, specialized epithelial cells called goblet cells secrete mucus into the lumen forming a bi-layered structure^16^. The inner mucus layer lies directly on top of the epithelium and acts as a barrier to microbes and the outer mucus layer is of loose composition and houses intestinal microbes^16^. The presence of intestinal microbiota is critical for the stimulation of mucus production as gnotobiotic mice have a thinner inner mucus layer^16^. Further, the composition of the microbiota is an important regulator of mucus production and specific microbial species have been shown to promote mucus production^17^,^18^,^19^,^20^. Members of the *Bacteroidetes* phylum, specifically *B. thetaiotaomicron*, are important colonizers and grazers of the outer mucus layer utilizing mucin-associated carbohydrates as an energy source^19^,^21^,^22^. Recently, a known mucin-degrading bacterium, which resides in the outer mucus layer called *Akkermansia muciniphila*, was shown to promote the production of the inner mucus layer^17^,^18^. It is likely that many other yet unidentified microbes inhabiting the intestine (bacteria, micro-eukaryotes and viruses) may also act to promote mucus secretion by goblet cells. Yet no studies to date have shown conclusive evidence of this. Microbial utilization of intestinal mucins results in degradation of mucin peptides and utilization of the associated *O*-linked glycans as an important energy source. Consequent to glycan utilization by certain microbes is the production of waste products, including short-chain fatty acids (SCFAs) butyrate, acetate, and propionate^23^,^24^. Butyrate production by microbes has been implicated in a feedback mechanism that is involved in upregulating mucus production by goblet cells in response to mucin utilization, replenishing the utilized mucins and associated glycans^25^,^26^. Through this positive feedback loop, certain microbes may promote host-mediated mucus secretion through the utilization of mucins and their associated carbohydrates as an energy source. Mucus production is an energy intensive process, and it is becoming clear that products of microbial metabolism, such as SCFAs, may provide the extra energy required for mucus generation in the intestine, as they can feed into the citric acid cycle.

In this study, we explore the ability of the phytonutrients anethol, carvacrol, cinnamaldehyde, and eugenol essential oils (the main components of fennel, oregano, cinnamon and clove, respectively), as well as capsicum oleoresin and garlic extract to modulate intestinal immune responses in mice. We show that eugenol treatment leads to increased resistance to enteric pathogen infection. Furthermore, we define a novel mode of action of eugenol that involves shifting the composition of the intestinal microbiota and stimulating mucus production, resulting in a thickening of the inner mucus layer.

## Results

### Phytonutrients alter gene expression in the colon

**Figure 1:**
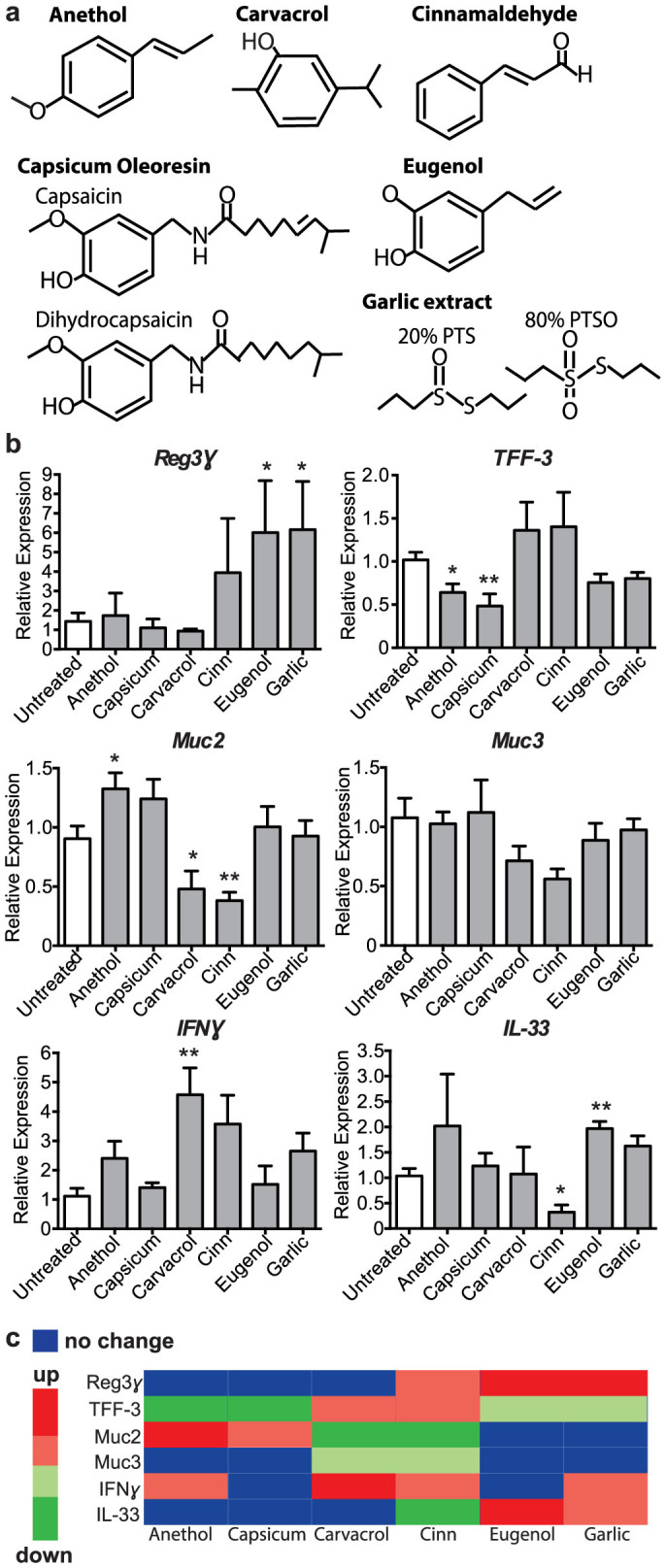
Phytochemical treatment results in pleiotropic effects on intestinal gene expression. (a), Chemical structures of the phytonutrients administered in the drinking water of mice. (b), Quantitative RT-PCR results shown of *Reg3γ* (*p = 0.0499; *p = 0.0340), *TFF-3* (*p = 0.0229; **p = 0.0074), *Muc2* (*p = 0.0421; *p = 0.0453; **p = 0.0092), *Muc3, IFNγ* (**p = 0.0075), and *IL-33* (*p = 0.0129; **p = 0.0036) expression relative to GAPDH in tissue from the distal colon of untreated and phytonutrient treated mice, n = 4 mice per group. Significance determined using two-tailed Student's t-test and expressed as the mean ± SEM. *p*-values listed as they occur in the graph left to right. Cinn = cinnamaldehyde; Capsicum = capsicum oleoresin. (c), Heat map showing the effect of phytonutrient administration to changes in colonic gene expression. Significant changes (*p* < 0.05) shown as bright green or red, trends shown as pale green or red, and no change shown with blue. Cinn = cinnamaldehyde; Capsicum = capsicum oleoresin.

We treated the drinking water of mice with one of six phytonutrients: anethol, anethol, carvacrol, cinnamaldehyde, eugenol, capsicum oleoresin and garlic extract (structures shown in Fig. 1a). After a 7-day treatment with each phytonutrient, we screened the expression of genes related to barrier function and innate immunity in the distal colon and found that each phytonutrient caused unique and pleiotropic changes in expression (Fig. 1b, c). The expression of *Reg3γ*, an antimicrobial peptide, was significantly promoted by both eugenol and garlic extract treatment (*P* = 0.0499 and *P* = 0.0340, respectively). Genes related to mucus production in the intestine were screened, including *Muc2*, *Muc3* and *TFF3*. Muc2 is the main secretory protein that makes up the mucus layer in the intestine, however, it is likely that Muc2 regulation occurs at the protein level due to its need for post-translational modification by glycosylation^24^. Muc3 is a membrane bound mucin protein, which aids barrier function and is upregulated during inflammation^27^,^28^. TFF3 is secreted by goblet cells and is an important antimicrobial mechanism which synergizes with Muc2 enhancing the protective properties of the mucus layer^29^. Surprisingly, the expression of many of these genes was reduced with phytonutrient treatment, with the exception of anethol treatment, which caused a significant increase in *Muc2* expression (*P* = 0.0421). IFNγ is a cytokine critical in type-1 innate immune responses acting as an important activator of macrophages resulting in potent immune responses against intracellular bacteria, viruses and tumors. We found carvacrol treatment significantly upregulated *IFNγ* expression (*P* = 0.0075); anethol, cinnamaldehyde, and garlic extract treatment also show trends of upregulation however these are not statistically significant (*P* = 0.0925, 0.0518, and 0.0615, respectively). In contrast, IL-33 is a cytokine that mediates type-2 innate immunity and is important in the activation of mast cells, eosinophils and basophils. Expression of *IL-33* was significantly reduced with cinnamaldehyde treatment (*P* = 0.0129), complementing cinnamaldehyde's function in promoting type-1 innate immunity via upregulation of *IFNγ* expression. In contrast, eugenol caused significant upregulation of *IL-33* (*P* = 0.0036) with garlic extract showing a trend in increased expression of *IL-33* (*P* = 0.0554).

### Eugenol treatment stimulates the production of the inner mucus layer

**Figure 2:**
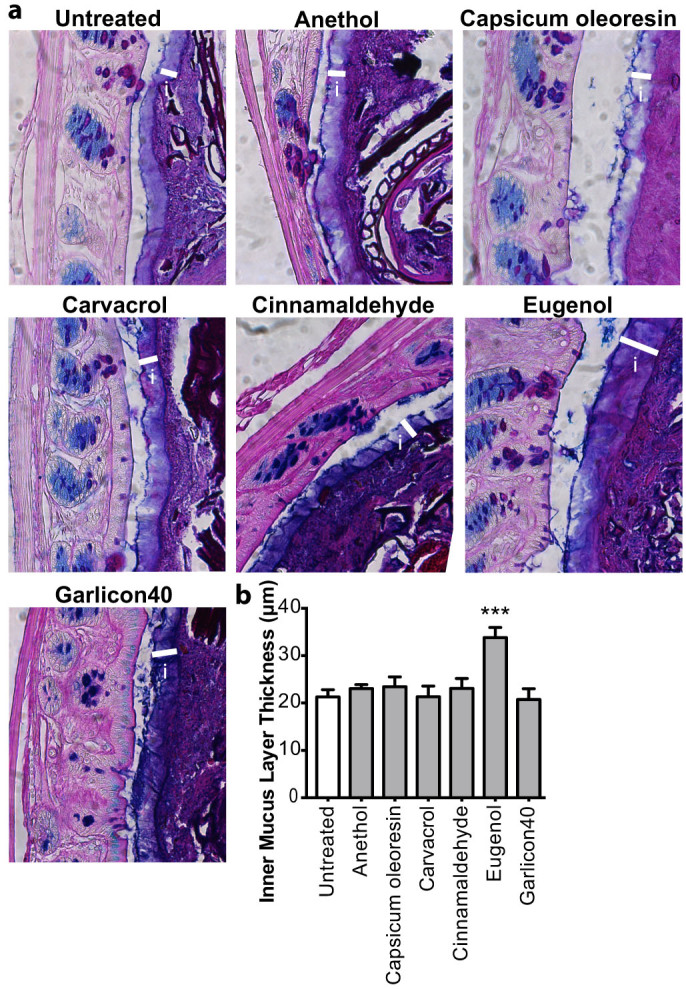
Eugenol treatment results in thickening of the inner mucus layer. (a), AB/PAS stained methanol-Carnoy's fixed distal colon sections showing the inner mucin layer (white bars). i = inner mucus layer. Original magnification = 400×. (b), Quantification of inner mucus layer thickness. Distal colon sections stained with AB/PAS to visualize and quantify the inner mucus layer. n = 5–10 mice per group. (*p = 0.038; ***p = 0.0003) U = untreated mice. Significance determined using two-tailed Student's t-test and expressed as the mean ± SEM.

The findings that phytonutrient treatment results in changes to goblet cell-specific gene expression, including *TFF-3* and *Muc2* (Fig. 1b), led us to hypothesize that phytonutrients may have an effect on the integrity of the inner mucus layer in the large intestine. To verify this we visualized the intestinal mucus layer by staining with AB/PAS and measured the inner mucus layer in the large intestine after 7 days of phytonutrient treatment (Fig. 2). None of the phytonutrients caused a reduction in inner mucus layer thickness, therefore preserving this important host defense (Fig. 2a). A striking increase in the thickness of the inner mucus layer was seen after eugenol treatment (Fig. 2a, b). Whereas the inner mucus layer thickness of untreated mice was on average 21 μm (+/− 4 μm), eugenol-treated mice exhibited a significantly thicker inner mucus layer, measured to be 34 μm (+/− 6 μm), an average increase of 59% compared to untreated mice (Fig. 2b). These data suggests that eugenol may enhance host defense through thickening of the inner mucus layer.

### Eugenol significantly alters the microbial composition in the intestine and microbial changes correlate with mucus production

**Figure 3:**
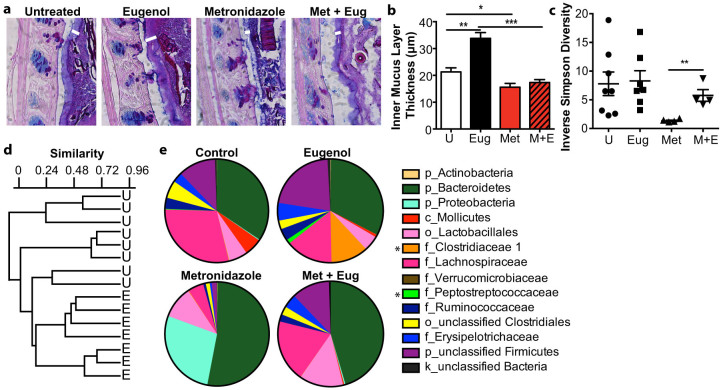
Eugenol treatment when combined with metronidazole prevents mucus thickening and highlights the importance of microbial factors. (a), AB/PAS stained methanol-Carnoy's fixed distal colon sections showing the inner mucin layer (white arrowheads). i = inner mucus layer. Original magnification = 400×. (b), Quantification of inner mucus layer thickness. Distal colon sections stained with AB/PAS to visualize and quantify the inner mucus layer. n = 5–10 mice per group. (*p = 0.038, ***p = 0.0003, ***p = 0.0001) U = untreated mice. Significance determined using two-tailed Student's t-test and expressed as the mean ± SEM. (c), Simpson's Reciprocal Index of diversity was used to determine diversity of fecal communities after treatment with eugenol, metronidazole, metronidazole and eugenol or untreated mice. n = 4–8. U = untreated mice. (**p = 0.0048). (d), Similarity between the microbiota of untreated and eugenol treated feces was measured by Bray-Curtis index and depicted in a dendogram. U = untreated, E = eugenol treated. (e), Family level phylogenetic classification of 16S rRNA gene frequencies in feces collected from untreated, eugenol, metronidazole or combined treatment. Those indicated with a classification level other than family level could only be identified confidently to the level indicated. Classification scheme: k, kingdom; p, phylum; c, class; o, order; f, family. Representative data is shown for each group, n = 4–8. Stars indicate significant family changes between eugenol and untreated mice (*p* < 0.05).

It is well established that mucus-secretion to homeostatic levels requires microbial signals in the intestine, as shown in gnotobiotic mice^16^,^30^,^31^. We have previously shown that treatment with the antibiotic metronidazole causes a reduction in goblet cell gene expression and thinning of the inner mucus layer and significant changes to the microbial community of the intestine^32^. We hypothesize that the drastic change metronidazole treatment causes to the intestinal microbiota may deplete the microbial signals required for production of the inner mucus layer. Thus eugenol may be utilizing a similar (but opposing) mechanism to increase the abundance of specific organisms to cause thickening of the inner mucus layer. Here we define the effects of metronidazole and eugenol treatment independently and in combination in terms of the thickness of the inner mucus layer and microbial composition of the intestine (Fig. 3). When eugenol treatment was combined with metronidazole treatment, no thickening of the inner mucus layer was observed and the mucus layer appears thinner and similar to metronidazole treatment alone (Fig. 3a, b). To correlate the inner mucus layer thickening effect of eugenol treatment with changes to the intestinal microbiota, fecal samples from eugenol, metronidazole, both metronidazole and eugenol treated, and untreated mice were assessed by pyrosequencing the V1–V3 regions of bacterial 16S rRNA genes. After quality filtering, the mean number of sequences (±SE) per group was 1100 ± 160. Microbial diversity, as indicated by Simpson's Reciprocal index (Fig. 3c) was similar in untreated and eugenol treated samples, with a striking reduction after metronidazole treatment. Remarkably, given the low dose of eugenol administered, combinational treatment of metronidazole and eugenol resulted in increased diversity compared to metronidazole alone (*P* < 0.005). Similarity between all samples was measured by Bray-Curtis metrics, where a value of 1 indicates identical samples and a value of 0 indicates no shared OTUs. Similarity between samples within the eugenol group (0.568+/−0.18) was higher (*P* < 0.001) as compared to the untreated mice (0.374+/−0.48), which may indicate a stabilizing effect on the community (Fig. 3d). At the family level eugenol treatment was sufficient to selectively increase the abundance of two bacterial families from the order *Clostridiales*, *Clostridiaceae 1* and *Peptostreptococcaceae* (Fig. 3e). Furthermore, we found that eugenol-dependent increase in these families correlated significantly with increased thickness of the inner mucus layer (Fig. 3a, b, e). Bacteria from these families were depleted with metronidazole treatment and could not be rescued when eugenol was added to the metronidazole treated mice (Fig. 3a, e). The combined metronidazole and eugenol treatment also resulted in increased microbial diversity when compared to metronidazole alone (Fig. 3e).

**Figure 4:**
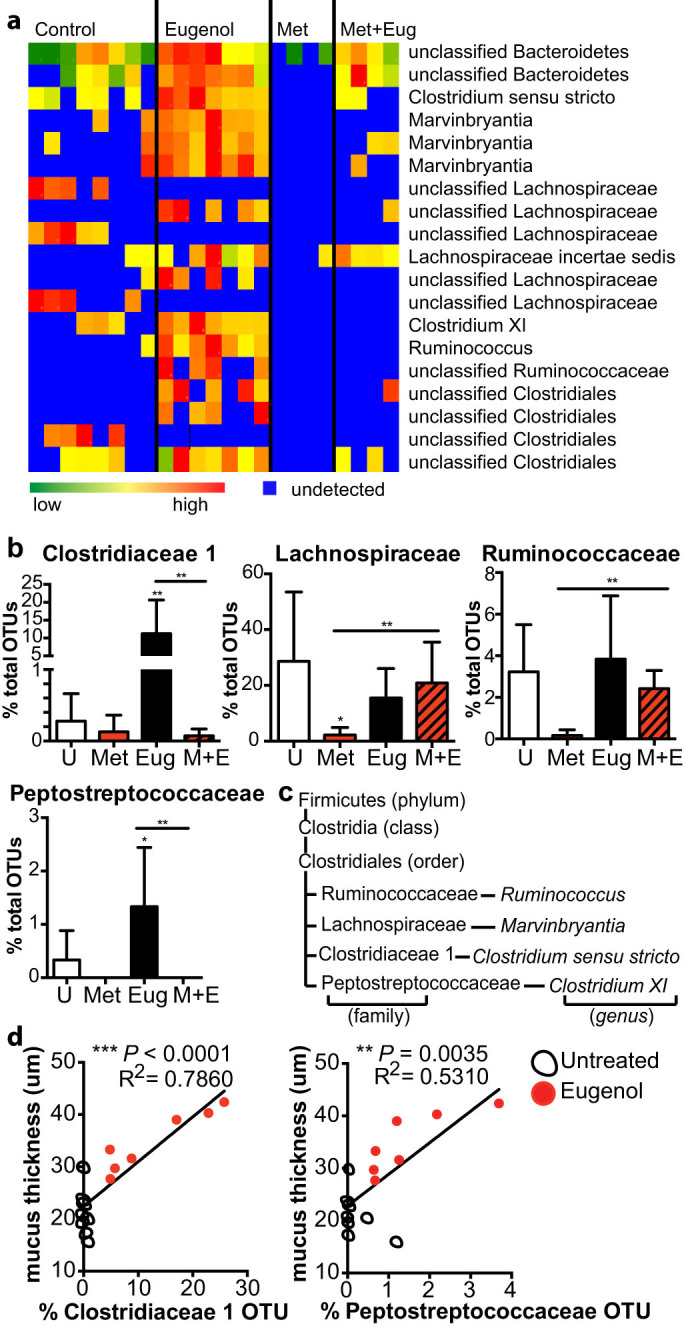
Eugenol specifically increases the abundance of the *Clostridiaceae 1* and *Peptostreptococcaceae* families. (a), Heatmap showing the relative abundance of fecal bacterial OTUs that differed between mice treated with eugenol, metronidazole, combination of both or untreated mice (*P* < 0.05). Classification scheme: p, phylum; c, class; o, order; f, family; g, genus. (b), Abundances of OTUs that increased with eugenol treatment, including the *Clostridiaceae 1* (Eug: **p = 0.0016, Eug vs. Met + Eug: **p = 0.0062), *Lachnospiraceae* (*p = 0.0127, **p = 0.0043), Peptostreptococcaceae (*p = 0.0127, **p = 0.0043), and *Ruminococcaceae* (**p = 0.0043) families. Data represented as percent of total OTUs. U = untreated mice. M + E = metronidazole and eugenol treated mice. (c), The miniature phylogeny tree shows the specific families belonging to the *Clostridiales* order and any relevant genera whose abundance is increased with eugenol treatment. Brackets indicate the classification. (d), Linear regression analysis showing the correlation of mucus thickness with abundance of *Clostridiaceae* or *Peptostreptococcaceae*.

Although eugenol treatment did not have a significant effect on the diversity of the intestinal community compared to untreated mice, it did increase the abundance of specific operational taxonomic units (OTUs) classified within the *Clostridiales* order, including members of the *Lachnospiraceae, Ruminococcaceae, Clostridiaceae 1* and *Peptostreptococcaceae* families (Fig. 4b). To deduce which of these families of bacteria were driving changes in mucus thickness, we looked at differences in OTU abundance in each of the treatment groups. *Clostridiaceae 1* was dominated by a single OTU classified in the *Clostridium sensu stricto* genus and represented a large component of the eugenol treated microbiota (11.2% ± 3.3), whereas it was at relatively low abundance in untreated mice (0.4% ± 0.1) (Fig. 4c). The *Peptostreptococcaceae* family was also dominated by a single OTU, which was classified in the genus *Clostridium* cluster *XI*, however the increase was less substantial (1.1% ± 0.4 in eugenol vs. 0.6% ± 0.2 in untreated) (Fig. 4c). The *Lachnospiraceae* and *Ruminococcaceae* families were increased with eugenol treatment alone and the combined treatment of eugenol and metronidazole (Fig. 4c). This increase in abundance did not coincide with increased mucus production as the combined metronidazole and eugenol treatment does not result in thickening of the inner mucus layer (Fig. 3a, b). The increase in abundance of *Clostridiaceae 1* with eugenol treatment correlates well with the increased inner mucus layer thickness and regression analysis shows an R-squared value of 78.6% (*P* = 0.0003) (Fig. 4d). The abundance of the *Peptostreptococcaceae* family shows a weak correlation to the thickness of the inner mucus layer with an R-squared value of 53.1% (*P* = 0.0035) (Fig. 4d), whereas the *Lachnospiraceae* and *Ruminococcaceae* families do not correlate with inner mucus layer thickness with R-squared values of 0.03% and 5.4%, respectively (data not shown). The inability of eugenol to induce thickening of the inner mucus layer in the presence of metronidazole further supports the potential role of the *Clostridiaceae 1* and *Peptostreptococcaceae* families in inducing mucus secretion as metronidazole treatment targets these bacteria preventing their colonization of the intestine during the combined treatment (Fig. 4c).

### Eugenol treatment confers resistance to *C. rodentium* infection and reduces systemic inflammation

**Figure 5:**
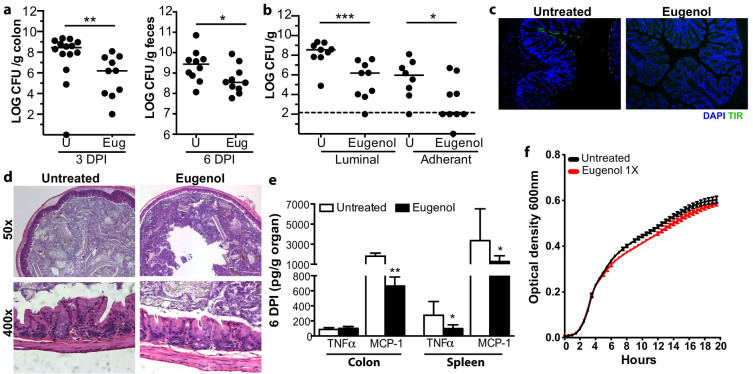
Eugenol treatment reduces *C. rodentium* burden in the colon. (a), Enumeration of *C. rodentium* in the colon and feces at day 3 and day 6 p.i., respectively. Each data point represents one individual. Results are pooled from two separate infections, n = 10–13 per group. Significance determined using the Mann-Whitney U-test and expressed as the median. U = untreated mice. (**p = 0.0048; *p = 0.0431). (b), Both luminal (fecal matter) and adherent (extensively washed colons) bacterial colonization is reduced in eugenol treated mice at day 3 p.i. Results are pooled from two separate experiments, n = 9 per group. Significance determined using the Mann-Whitney U-test and expressed as the median. (*p = 0.05; ***p < 0.0008) U = untreated mice. Dashed line represents detection limit. U = untreated. (c), Representative immunostaining for the *C. rodentium*-specific effector Tir (green) in colon, with DAPI (blue) as a counter stain at 3 days p.i.. Tir staining present at day 3 p.i. in untreated mice which is absent in eugenol treated mice. Original magnification = 100×. (d), H&E stained distal colon sections from untreated and eugenol treated mice at day 3 p.i. Minor inflammation is evident in untreated and eugenol treated mice (top panel original magnification = 50×; bottom panel original magnification = 400×). (e), Cytokine and chemokine production, TNFα and MCP-1, in the colon and spleen in response to *C. rodentium* infection 6 days p.i. Results shown as pg of cytokine produced per gram of organ, n = 9–12 mice per group. (**p = 0.0092; TNFα *p = 0.0172; MCP-1 *p = 0.0351) Significance determined using two-tailed Student's t-test and expressed as the mean ± SEM. (f), *C. rodentium* was grown with or without eugenol at 13.3 ug/mL (denoted 1×) and growth was determined using optical density measurements every hour for 20 hours at 600 nm.

The inner mucus layer is known to be an important host defense to enteric pathogens, including the attaching and effacing pathogen, *C. rodentium*^32^,^33^. Genetic mutations that result in abrogated mucus secretion result in exacerbated disease following *C. rodentium* infection^27^,^33^,^34^,^35^,^36^,^37^. As eugenol treatment results in thickening of the inner mucus layer, we investigated whether this would be sufficient to confer increased colonization resistance to *C. rodentium*. Strikingly, eugenol treated mice showed decreased *C. rodentium* burdens in the colon both at 3 days post infection (p.i.) and in the feces at 6 days p.i. (Fig. 5a). We then investigated the rate of attachment of *C. rodentium* by determining the luminal (fecal associated) and the adherent (attached to the intestinal epithelium) burdens early in infection, at 3 days p.i.. Eugenol treatment led to decreased attachment of *C. rodentium* at 3 days p.i. (Fig. 5b). To visualize the extent of *C. rodentium* attachment, we performed immunostaining for the *C. rodentium*-derived infection marker Tir (translocated intimin receptor) on colon sections 3 days p.i.. Eugenol treatment inhibits *C. rodentium* attachment to the epithelium (Fig. 5c), resulting in significantly lower numbers of pathogen burden at the early stages of infection (Fig. 5b). Histological analysis of the colon early in infection (3 days p.i). showed no major pathological changes in both untreated and eugenol treated mice (Fig. 5d). Later in infection (6 days p.i.), systemic inflammation was dampened in eugenol-treated mice, which showed reduced TNF-α and MCP-1 levels in the spleen (Fig. 5e). Eugenol did not have a direct antimicrobial effect on *C. rodentium* as growth was unaltered in Luria broth supplemented with eugenol at the concentration given to mice (Fig. 5e). At the dose given in the drinking water of mice (denoted by 1×), eugenol did not affect growth in LB (Fig. 5e).

## Discussion

In this study we have shown that after only 7 days of phytonutrient treatment there are dramatic changes in gene expression related to mucosal homeostasis. Specifically, we found that eugenol treatment results in thickening of the inner mucus layer. Further we characterized the impact eugenol had on the intestinal microbial community and found after Bray-Curtis analysis, the microbiota of eugenol treated mice clustered away from the wild-type controls, implying a significant change in community composition. The most striking difference was seen in the order *Clostridiales*, which were enriched following eugenol treatment, and notably absent in metronidazole treated mice, which exhibit a significant thinning of the inner mucus layer. Thus, eugenol may mediate this mucus thickening effect by increasing the abundance of specific genera within the *Clostridiales* order.

In our initial gene expression screen, the changes we note suggest that anethol, carvacrol, cinnamaldehyde, eugenol, capsicum oleoresin and garlic extract act by different mechanisms and may function through multiple direct and/or indirect mechanisms. These phytonutrients may act directly on host intestinal cells to strengthen mucosal defenses through the upregulation of antimicrobial peptides, including Reg3γ. We found eugenol treatment to significantly upregulate Reg3γ expression and this may mediate some of eugenol's antibacterial effects and further explain how eugenol changes the architecture of the microbiota. Additionally, these phytonutrients may act directly on host mucosa to maintain the integrity of the mucus barrier in the intestine by regulating mucus layer associated proteins, including Muc2 and Muc3, and intestinal trefoil factor (TFF3). Expression levels of the cytokines IFNγ and IL-33 are also modulated following phytonutrient treatment, which suggests that the phytonutrients may influence innate immune pathways. Alternatively, phytonutrients could be acting indirectly by causing changes to the microbial architecture of the intestine as we described for eugenol treatment. Changes in intestinal microbial communities are known to cause downstream effects on host immunity and susceptibility to various diseases, including enteric infections^38^,^39^,^40^.

This study characterizes a novel immunomodulatory role of eugenol as a compound that can promote mucus secretion and formation of the inner mucus layer in the large intestine. Eugenol is a member of the phenylpropanoids class of chemical compounds and can be extracted from the essential oil of cloves. Historically eugenol has been used to treat dental problems due to its analgesic, anesthetic, anti-inflammatory and antibacterial effects^41^,^42^. Previous studies have shown that eugenol is absorbed into the tissues of rats, including the intestine^43^,^44^. Given this, we appreciate the possibility that the effects we describe of diet supplementation with eugenol may be a direct interaction with intestinal tissue stimulating mucus secretion, independent of the microbial changes we characterize. However, since the impact of eugenol on mucus-stimulation is abrogated by an antibiotic, the mechanism driving eugenol's effect is, at least in part, mediated by the intestinal microbiota. Perhaps eugenol acts through a combination of factors, both host tissue stimulation and microbial changes, to mediate it's protective effects. Future studies to assess the ability of eugenol to function as a mucus secretagogue independent of the intestinal microbiota could be done to determine the tissue specific effects of eugenol.

We have also identified two families of bacteria, *Clostridiaceae 1* and *Peptostreptococcaceae*, which may mediate the effect of eugenol treatment on mucus production. Both of these families increased in abundance with eugenol treatment alone but did not increase in abundance with the combined treatment of metronidazole and eugenol (which does not result in mucus thickening compared to eugenol alone). This combined treatment of metronidazole and eugenol allowed recognition of the microbial changes that occur with eugenol treatment that are not related to thickening of the inner mucus layer. Eugenol was found to increase the abundance of the *Lachnospiraceae* and *Ruminococcaceae* families both in the absence and presence of metronidazole, suggesting these two families are not involved in the increased inner mucus layer production. Previous studies have found that other members of the *Clostridiales* order, including *Clostridium perfringens* and *Clostridium septicum*, exhibit mucinase activity and can utilize associated oligosaccharides as energy sources^45^,^46^. Although no information of the mucinase activity of *Clostridium sensu stricto* and *Clostridium* cluster *XI* is known, we would hypothesize based on our data that these genera may also utilize mucins as energy sources. Eugenol, by increasing the abundance of these genera, would cause an increase in microbe-mediated mucin utilization and a subsequent increase in the production of short-chain fatty acids (SCFA) causing the host to respond with increased production, subsequently thickening the inner mucus layer. Similarly, this has been previously shown with *Bacteroides thetaiotaomicron*^20^, the byproducts of this nutrient utilization results in an increase of bacterial waste products, such as SCFA, that can stimulate the intestinal epithelium to produce more mucus^20^,^31^. Members of the Clostridia class have been shown to be closely associated with the host mucosa suggesting that these bacteria may be critical regulators of mucus production by the host^47^,^48^. With the increase in knowledge of the health benefits of certain Clostridia species there still lacks specific therapies that can effectively deliver or upregulate these members in the human intestinal microbiota. Probiotic therapeutics delivering these species would be difficult to cultivate and package on a large scale, as this would have to be done in an anaerobic environment. Eugenol diet supplementation may be of great interest as an alternative and more practical method to therapeutically result in an increase of Clostridia species in the human microbiota.

The changes in microbial composition and increases in mucus secretion due to eugenol treatment are functionally relevant to infection, as they correlated with heightened colonization resistance to *C. rodentium*. Reduced colonization is likely not a consequence of bactericidal effects of eugenol as growth of *C. rodentium* was unchanged in Luria broth at the same dose used to treat mice. This decreased colonization was due to inhibited attachment of *C. rodentium* to the surface of the intestinal epithelium. Concordant with our previous studies showing thinning of the inner mucus layer results in an increased rate of attachment by *C. rodentium*^32^ and it is probable that an increase in the thickness of the inner mucus layer would delay pathogen adherence. We have also observed this in *in vitro* studies showing delayed EPEC adherence to a mucus-producing T84 colonic adenocarcinoma cell line after treatment with a calcium ionophore (data not shown), a known mucus secretagogue, and also shown previously by McCool *et al*^49^. Further, the probiotics *Lactobacillus plantarum* 299 v and *Lactobacillus rhamnosus* have been shown to upregulate mucus production, specifically Muc2 and Muc3, inhibiting EPEC adherence to HT-29 intestinal epithelial cells^50^. Probiotics have been touted as replacements to antibiotic growth promoters by inducing a healthier intestinal environment through innate immune induction and pathogen exclusion^15^. However, the literature regarding the use of probiotics show variable efficacy and several factors can interfere with their universal use, such as the colonization ability of the probiotic in different animals, mechanism of action (can be host dependent, with significant variability), as well as its stability in feed. The results presented here demonstrate that low doses of phytochemicals, specifically eugenol, could provide a working alternative or be used in conjunction with probiotics to improve intestinal health, promote microbial changes, and reduce enteric pathogen burden.

This study provides the first evidence that a phytonutrient given at a low dose can significantly alter the architecture of the colonic microbiota, which may mediate the resultant thickening of the inner mucus layer. The low dose treatment was able to change the configuration of the microbiota selectively increasing the abundance of primarily the Clostridiales order, including members of the genera *Marvinbrytantia, Ruminococcus*, *Clostridium sensu stricto* and *Clostridium* cluster *XI*. We also showed that eugenol treatment was able to inhibit adherence of *C. rodentium* to the intestinal epithelium early in infection, a likely consequence of a thicker inner mucus layer. Future studies should focus on the interaction of eugenol with *Clostridium sensu stricto* and *Clostridium* cluster *XI* and the role these species have in promoting the mucus barrier.

## Methods

### Mice

All animal work was done according to the Canadian Council on Animal Care guidelines, utilizing protocols that were approved for use by the Animal Care Committee at the University of British Columbia (UBC). 8 to 10 week old C57BL/6 female mice (Jackson Laboratory, Bar Harbor, ME) were housed in the animal facility at UBC. Mice were fed a standard sterile chow diet (Laboratory Rodent Diet 5001, Purina Mils, St. Louis, Missouri) *ad libitum* throughout experiments. Phytonutrient treated mice were given 13.3 μg/mL of the phytonutrient in drinking water for 7 days. This low concentration is consistent with the 3–5 g/metric ton of the phytonutrients used in livestock feed. Anethol, carvacrol, cinnamaldehyde and eugenol were synthetically produced at 95% purity and identical to the natural compound. Capsicum oleoresin is a standardized natural extract with 6% capsaicin and dihydrocapsaicin. Garlic extract is a botanical extract from garlic, standardized to 40% propyl thiosulfonates. This 40% is composed of greater than 80% propyl propane thiosulfonate (PTSO) and the remainder as propyl propane thiosulfinates (PTS). All phytonutrients were produced and provided by Pancosma (http://www.pancosma.com/). Antibiotic treated mice were given metronidazole (Sigma) at 750 mg/L in drinking water for 4 days. Untreated, control, mice received sterilized water. After 7 days of initial phytonutrient treatment, mice were infected with *Citrobacter rodentium* and phytonutrient treatment was continued for the duration of the infection or mice were euthanized and tissues harvested.

### RNA isolation, cDNA synthesis, and real time polymerase chain reaction

The terminal 2–3 mm of the colon were excised, immediately submerged in RNA*later*™ (Qiagen) and stored at 4°C overnight and then at −80°C for subsequent RNA extraction. RNA was extracted using RNeasy Mini kit (Qiagen) according to the manufacturer's instructions. RNA concentration was determined using a NanoDrop ND-1000 (NanoDrop Technologies, Wilmington, DE, USA) with A260/A280 ratio's for all samples analyzed between 2.0–2.1. RNA integrity of each sample was determined using a Bioanlayzer RNA 6000 nano and the average RNA integrity number (RIN) was 9.7 (range 7.3–10). Reverse transcription was performed with the Quantitect RT kit (Qiagen) using the manufacture-provided mix of oligo-dT and random primers for cDNA synthesis and adding 1 μg RNA as template. A genomic DNA wipeout step was preformed at 42°C for 2 min followed by reverse transcription at 42°C for 30 min and 95°C for 3 min. cDNA was diluted 1:10 prior to real-time PCR.

Real-time PCR was performed using Quantitect SYBR-Green Mastermix (Qiagen) and with the primers listed in Table 1. Both Reg3γ and IL-33 primers were purchased from Qiagen (catalogue number: QT00147455 and QT00135170, respectively). PCR was performed on an Opticon 2 (Bio-Rad) in a 10 uL total reaction volume and cycles consisted of 95°C for 15 min and 40 cycles of 95°C for 15 s, 60°C for 30 s and 72°C for 30 s. Glyceraldehyde-phosphate-dehydrogenase (GAPDH; NM_001289726.1) was found to be an appropriate endogenous control and was used for normalization. The relative expression was calculated using the ddCt method corrected for primer efficiencies (listed in Table 1) according to Pfaffl^51^.

### Measurements of mucus thickness ex vivo

From the distal colon, 5 mm of tissue containing a fecal pellet was excised, immediately submerged in Methanol–Carnoy's fixative at 4°C for 2 hours and then placed into 100% ethanol. Fixed colon tissues were embedded in paraffin and cut into 5 μm sections and subjected to Alcian blue periodic acid-Schiff (AB/PAS) staining. The inner mucus width was determined by an average of 5–10 measurements per field with 4 fields counted per tissue section.

### Microbial analysis

For composition analyses, total DNA was extracted from fecal pellets collected from untreated, phytonutrient or antibiotic treated mice using the Ultra Clean Fecal DNA kit (Mo Bio Laboratories, Carlsbad, CA), including physical disruption using a FastPrep instrument (MP Biomedicals, Solon, OH) for 60 sec at level 5. 16S rRNA gene fragments were PCR amplified with nucleotide-bar-coded primer pairs 27F: 5′-AGAGTTTGATCMTGGCTCAG-3′and 510R: 5′-GWATTACCGCGGCKGCTG-3′. PCR products were gel-purified (QIAquick gel extraction kit, Qiagen, Valencia, CA). Each amplicon (100 ng) was pooled and pyrosequenced using a 454 Titanium platform (Roche, Branford, CT).

### Bioinformatics

Sequences were processed using MOTHUR according to the standard operating as previously described, accessed on July 10, 2013^52^. Quality sequences were obtained by removing sequences with; ambiguous bases, quality read length less than 200 bases and chimeras identified using chimera.uchime. Quality sequences were aligned to the silva bacterial reference alignment and operational taxonomic units (OTU) were generated using a dissimilarity cutoff of 0.03. Sequences were classified using the classify.seqs command with RDP as reference. Inverse Simpson's diversity index was used to calculate diversity. The Bray Curtis index was used as a measure of similarity in microbial composition between samples. Diversity, similarity and abundance of bacterial OTUs and families were compared using the Mann-Whitney *U*-test or student's T-test. Bonferroni correction was applied in cases of multiple comparisons.

### Bacterial strains and infection of mice

Mice were infected by oral gavage with 0.1 mL of an overnight culture of LB containing approximately 2.5 × 10^8^ colony forming units (CFU) of a streptomycin-resistant derivative of *C. rodentium* (DBS100).

### Citrobacter rodentium CFU and cytokine determination

The colon was excised and colonic tissue was separated from fecal contents, the tissue was extensively washed in sterile PBS. Whole spleen, feces and colon tissues were collected in 1 mL of sterile PBS supplemented with complete ethylenediaminetetraacetic acid-free protease inhibitor cocktail (Roche Diagnostics) at a final concentration recommended by the manufacturer. Tissues were weighed, homogenized in a MixerMill 301 bead miller (Retsch) for 5 minutes at 30 Hz at room temperature. Tissue homogenates were serially diluted in PBS and plated onto MacConkey Agar (Difco), incubated overnight at 37°C, and bacterial colonies were enumerated the following day, normalizing them to the tissue weight (per gram). *C. rodentium* colonies were clearly identified by their unique characteristic of being round with red center and a white rim. Colon and spleen homogenates were centrifuged twice at 15,000 *g* for 20 min at 4°C to remove cell debris, and the supernatants were aliquoted and stored at −80°C. Cytokine levels in colon and spleen homogenates were determined with the BD Cytometric Bead Array Mouse Inflammation Kit (BD Biosciences), according to the manufacturer's recommendations, and normalized to tissue weight (per gram).

### Immunohistochemistry

From the distal colon, 5 mm of tissue was excised and fixed in 10% neutral buffered formalin overnight and then placed into 70% ethanol. Fixed tissues were embedded in paraffin and cut into 5 μm sections then deparaffinized and rehydrated. Antigen retrieval was performed prior to blocking and staining by placing deparaffinized, rehydrated slides in 10 mM citric acid pH 6.0 at 90–100°C for 20 min, followed by cooling to room temperature. Immunostaining was carried out using antibodies against Tir (antibody production described before (14)) antibody at 4°C overnight followed by incubation with an Alexa488-conjugated secondary antibody (Invitrogen) for 1 h at room temperature. Tissues were mounted using ProLong Gold Antifade (Molecular Probes/Invitrogen) that contains 4′,6′-diamidino-2-phenylindole (DAPI) for DNA staining.

### Histopathological scoring

Tissues were fixed in 10% neutral buffered formalin overnight and then placed into 70% ethanol. Fixed tissues were embedded in paraffin and cut into 5 μm sections. Tissues were stained with hematoxylin and eosin (H&E), using standard techniques by the UBC Histology Laboratory. Tissue sections were assessed for pathology in four regions: lumen, surface epithelium, mucosa and submucosa.

### Statistical analysis

Statistical significance was calculated by using a two-tailed Student's *t-test* unless otherwise stated, with assistance from GraphPad Prism Software Version 4.00 (GraphPad Software, San Diego California USA, www.graphpad.com). If not otherwise specified statistical significance was given as *** *P*-value < 0.001; ** *P*-value < 0.01; * *P*-value < 0.05; ns (not significant) *P*-value > 0.05. The results are expressed as the mean value with standard error of the mean (SEM), unless otherwise indicated.

## Author Contributions

M.W. conceived the project, preformed experiments, interpreted the results, and wrote the manuscript. B.P.W. preformed all the analysis and interpretation of the intestinal microbiome data. D.M.B. helped conceive the project and provided the isolated phytonutrients. B.B.F. helped conceive the project and mentored and supervised its participants.

## Figures and Tables

**Table 1:** Primer sequences and efficiency (E) for host gene expression analysis

Gene	Target	Primer	Sequence	Product Length	E
Muc2	NM_023566.3	MUC2-F	GCTGACGAGTGGTTGGTGAATG	135 bp	2.045
		MUC2-R	GATGAGGTGGCAGACAGGAGAC		
Muc3	XM_006504541.1	MUC3-F	CGTGGTCAACTGCGAGAATGG	112 bp	2.042
		MUC3-R	CGGCTCTATCTCTACGCTCTCC		
IFNγ	NM_008337.3	IFNγ-F	TCAAGTGGCATAGATGTGGAAGAA	92 bp	2.124
		IFNγ-R	TGGCTCTGCAGGATTTTCATG		
TFF3	NM_011575.2	TFF3-F	CCTGGTTGCTGGGTCCTCTG	133 bp	1.998
		TFF3-R	GCCACGGTTGTTACACTGCTC		
IL-33	NM_001164724 NM_133775	Mm_Il33_1_SG QuantiTect Primer Assay		122 bp	2.141
Reg3γ	NM_011260	Mm_Reg3g_1_SG QuantiTect Primer Assay		93 bp	1.915
GAPDH	NM_001289726.1	GAPDH-F	ATTGTCAGCAATGCATCCTG	102 bp	2.099
		GAPDH-R	ATGGACTGTGGTCATGAGCC		
